# Dietary Vitamin E Isoforms Intake: Development of a New Tool to Assess Tocopherols and Tocotrienols Intake in Adults

**DOI:** 10.3390/nu15173759

**Published:** 2023-08-28

**Authors:** Kacper Szewczyk, Magdalena Górnicka

**Affiliations:** Department of Human Nutrition, Institute of Human Nutrition Sciences, Warsaw University of Life Sciences (SGGW-WULS), 02-776 Warsaw, Poland; kacper_szewczyk@sggw.edu.pl

**Keywords:** vitamin E, dietary intake, dietary assessment, adequate intake

## Abstract

Due to the documented health benefits of tocopherols and tocotrienols as bioactive compounds, it seems important to assess their intake. The aim of this study was to develop a new tool and its application for assessment of tocopherol and tocotrienol intake in adults. Dietary data were collected by semiquantitative FFQ (VitE-FFQ) and by a 1-day dietary record in a group of 447 subjects. The database of the US Department of Agriculture (USDA) was used to calculate the individual isoforms of vitamin E and develop the tool—VIT_E.CAL. The assessment of measuring agreement between the two methods was conducted by analysis of the correlations and Bland–Altman plots. The average α-tocopherol intake was 11.3 mg/day for the data obtained using the FFQ method and 12.8 mg/day for the results obtained using the 1-day dietary record. Depending on the adopted recommendation, only 40–57% of the subjects had adequate vitamin E intake. The intake of α-tocopherol did not exceed the UL value in any of the respondents. The dominant forms of vitamin E in the diet of the studied group were α- and γ- forms (55% and 38% of the total sum) among tocopherols and β- and γ- forms (49% and 24% of the total sum) among tocotrienols. VIT_E.CAL allows us to calculate not only the total amount of vitamin E but also its eight isoforms. It can be a useful tool to assess individual and group intake of various forms of vitamin E in the diet. The use of VIT_E.CAL enables the proper assessment of vitamin E (as α-tocopherol and not α-tocopherol equivalent) in the diet of Poles, and most likely also in the European diet. The obtained results indicate the need to take into account the content of individual forms of vitamin E in food/diet, which will allow for a reliable assessment of its consumption. It also seems necessary to standardize the nomenclature regarding the name of vitamin E and its use for correct nutritional assessment.

## 1. Introduction

Tocopherols and tocotrienols belonging to the vitamin E family are antioxidants and play specific roles in the human body [1,2]. There are eight different natural forms in the vitamin E family: α-, β-, γ-, and δ-tocopherols and α-, β-, γ-, and δ-tocotrienols [3]. The most biologically active form of vitamin E is α-tocopherol because of the presence of a specific α-tocopherol transfer protein (α-TTP). So far, α-TTP is the only known protein that precisely recognizes α-tocopherol in the liver and transfers it to lipoproteins for further circulation in the blood [4]. A mutation in the gene encoding the α-TTP protein can lead to the development of ataxia with vitamin E deficiency (AVED) [5]. The assessment of the human requirement for α-tocopherol is challenged by the rare occurrence of clinical symptoms of deficiency. Symptoms usually appear in premature babies, infants, and adults with fat malabsorption, liver disease, or genetic conditions [6]. The recommended dietary allowance (RDA) of vitamin E varies between countries, depends on age and gender, and these values often differ from each other. Current research shows that there is a constant need to review, define, and assess the dietary requirements of different populations for vitamin E or only α-tocopherol [7,8]. The US National Institute of Health (NIH) and US Institute of Medicine (IoM), based on evidence from clinical trials, recommend the intake of α-tocopherol for adults at the level of 15 mg per day (RDA) regardless of gender [9,10]. Many health policy makers, like the Nordic Council of Ministers [11], European Food Safety Authority (EFSA) [6], French Agency for Food, Environment, and Occupational Health and Safety (ANSES) [12], recommend intake at the level of 7.5–10 mg α-tocopherol/day. Polish recommendations have been developed by the National Institute of Public Health—National Institute of Hygiene—National Research Institute (NIZP–PZH–PIB) [13] as the adequate intake (AI) expressed in α-tocopherol equivalents, which takes into account the biological activity of all eight isoforms, at the level of 8 mg per day for women and 10 mg for men. Our national AI values [13] are slightly lower than those of the European Food Safety Authority (EFSA) [6], which are, respectively, 13 mg of α-tocopherol equivalent per day and 11 mg per day for men and women. The upper level intake (UL) for vitamin E has been set at 300 mg α-tocopherol per day for adults [6,13].

Tocopherols and tocotrienols neutralize free radical scavengers in membranes and lipoproteins [8]; of these isoforms, α-tocopherol is the most studied [14]. It is used in research on diseases with increased inflammation, mainly cancer, and diseases of the central nervous system, the immune system, and the cardiovascular system [15,16,17,18]. In recent years, other forms of vitamin E have been shown to have better antioxidant and anti-inflammatory properties than α-tocopherol, and research started focusing on these forms [19]. Much attention is also paid to γ-tocopherol, which is the dominant form of vitamin E in the US diet. γ-tocopherol shows higher activity in capturing reactive oxygen and nitrogen species than α-tocopherol, and its concentration in the blood may be related to a lower risk of cancer and cardiovascular diseases [19]. Research on tocotrienols is expanding worldwide due to their interesting biological properties that tocopherols do not have, including neuroprotective, radioprotective, anticancer, anti-inflammatory, and lipid-lowering properties [20]. Tocotrienols are also credited with angiogenic effects and participation in the regulation of the activity of enzymes and transcription pathways, which may be reflected in the prevention of cancer [21].

There are studies indicating the possible health benefits of consuming different forms of vitamin E [22,23,24,25]. The main food sources of tocopherols and tocotrienols are the lipid components of oilseeds and nuts. Sunflower, rapeseed, corn, linseed, soybean, almond, peanut, and olive oils are the most abundant sources of tocopherols [26]. The main rich, natural sources of tocotrienols are palm oil, rice bran, wheat germ oil, coconut oil, and annatto seeds. An extract from the annatto seeds of the achiote tree (*Bixa orellana* L.) consists of 90% δ-tocotrienol and 10% γ-tocotrienol; palm oil, extracted from the reddish pulp of the fruits of the palm tree (*Elaeis guineensis*), consists mainly of 46% γ-tocotrienol and 22% α-tocotrienol [20,27,28].

To the best of our knowledge, no previous study in Europe has assess the intake of individual forms of vitamin E with different biological activities. Considering that vitamin E is contained in products that are often skipped in methods using current records, i.e., added fats, sauces, nuts, or oilseeds, it is important to include them when developing tools for nutrition assessment. Moreover, taking into account that only α-tocopherol has the vitamin property and can be called vitamin E [8], tools are needed to evaluate the different forms currently included as vitamin E.

The above conditions were an argument for the development of a tool that would allow us to assess the consumption of individual forms of vitamin E. The aim of this study was to develop a new tool for the assessment of tocopherol and tocotrienol intake in adults by (i) developing a semiquantitative food frequency questionnaire (VitE-FFQ), (ii) creating a database of tocopherol and tocotrienol content, (iii) providing a tool (VIT_E.CAL) for the calculation of vitamin E content, and (iv) applying it to the assessment of tocopherol and tocotrienol intake.

## 2. Materials and Methods

### 2.1. Dietary and Socidemographic Data

**Figure 1:**
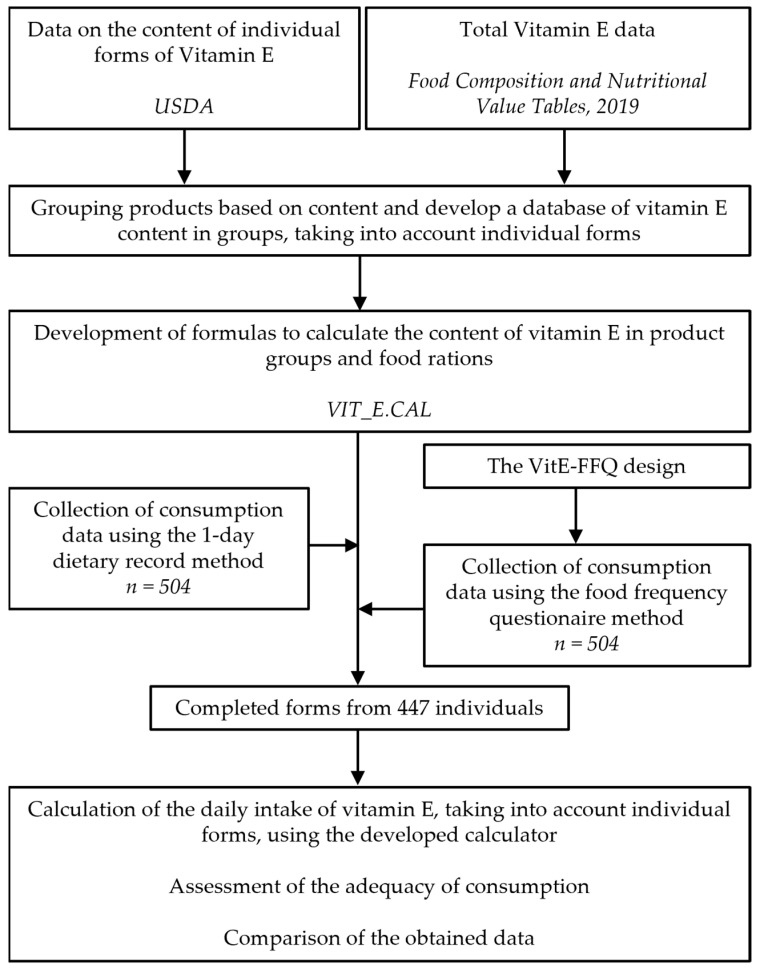
The design of the study.

This study was conducted for a period of 12 months, from September 2021 to September 2022, using random sampling in a group of adult Poles. During this period, participants were asked to complete the VitE-FFQ questionnaire and a 1-day dietary record immediately after completing the FFQ. Both the VitE-FFQ and the 1-day dietary record were self-reported. In addition, data on basic sociodemographics such as age, education, and place of living as well as the anthropometrics of body height and weight were collected. The inclusion criteria for this study were ages 18–65 and the use of a diet that did not eliminate any of the food groups. The exclusion criteria were gestation, vitamin E supplementation, and lack of or incomplete data about food consumption. The computer-assisted web-based interviewing (CAWI) method was used to collect all data. In this study, 504 participants took part. Finally, completed questionnaires from 447 respondents were included; 57 questionnaires were rejected due to nonfulfilment of the inclusion criteria and lack of data on consumption (Figure 1).

### 2.2. The VitE-FFQ Design

The developed semiquantitative FFQ to evaluate the consumption of tocopherols and tocotrienols in the diet with VitE-FFQ covers 8 groups of food products, such as vegetables, fruits and fruit products, legumes and legume products, nuts and oilseeds, fats, cereals, fish and fish products, and snacks and “others”. The VitE-FFQ questionnaire consists of a list of 67 food products that are sources of these compounds and their usual portion size according to the Polish Atlas of Portion Sizes of Food Products and Dishes [29]. The questionnaire was developed for assessing tocotrienol consumption in an initial study but has been improved for this investigation [30]. All food products were selected based on the content of vitamin E marked as the equivalent of α-tocopherol (Polish Food Composition and Nutritional Value Tables) [31]. In the developed VitE-FFQ, we asked respondents to indicate the usual number of servings of the listed products consumed in a week (Table 1). The questions concerned the 6-month period preceding the completion of the questionnaire. Moreover, in the information for the respondent, it was indicated that the respondent should recall all the ingredients of the dishes, such as the fats used or added nuts or oilseeds. The VitE-FFQ specifies that the usual number of servings should include portions of products eaten separately and added to prepared dishes, and it also indicates that not only whole numbers but fractions can be provided.

### 2.3. Development of the Tool Vit_E.CAL

The developed questionnaire VitE-FFQ, by taking into account the same products, is compatible with the calculator Vit_E.CAL. The tool was developed in MS Excel in 2 sheets (Supplementary Materials, VIT_E.CAL). Due to the fact that the Polish Food Composition and Nutritional Value Tables [31] only provide the total content of vitamin E expressed as the equivalent of α-tocopherol, the database of the United States Department of Agriculture (USDA) was used to calculate individual tocopherols and tocotrienols [32]. In the first sheet, “Product group database”, data on the content of tocopherols and tocotrienols were entered: in the rows, the products in each group were entered, while in the columns, the mean content of α-, β-, γ-, and δ-tocopherols and α-, β-, γ-, and δ-tocotrienols was entered. The added column “vitamin E added” refers to the amount of vitamin E added to the product by the manufacturer (data from the USDA database). The initial selection of products was made on the basis of the Food Composition and Nutritional Value Tables [31], and then this database was extended to include other products from the USDA database which are not included in the Food Composition and Nutritional Value Tables. Food products were organized into groups based on similar content of individual forms of vitamin E, where possible. Food products were grouped into the following categories: vegetables, fruit and fruit products, legumes and legume products, nuts and oilseeds, fats, cereals, fish and fish products, and snacks and others.

The second sheet, “VIT_E.CAL”, allows us to calculate the daily intake of individual forms of vitamin E. After entering information on the average number of servings of each group consumed during the week in the column “Number of portions”, the total amount of this product per week (column “Product consumption per 7 days [g]”) is calculated. The average daily consumption of the product in these groups is computed next (by dividing by the number of days—column “Average product consumption [g]”). Based on that, in the appropriate columns, the daily content of tocopherols and tocotrienols in the consumed product (mg) is calculated from the “Product group database” sheet. In the rows with the names of food product categories, formulas have been entered that allow the calculation of the sum of individual tocopherols consumed in a given product category. To simplify, the calculator uses the following procedure to calculate the daily intake of tocopherols and tocotrienols from particular groups of food products: consumption of the selected form of tocopherols or tocotrienols (mg) = daily number of portions x average content of the selected form of tocopherols or tocotrienols in one portion of a group of food products. It is also possible to calculate the sum of all forms of vitamin E as mg of α-tocopherol equivalents as follows:

Vit. E [mg α-tocopherol equivalents/100 g] = mg α-tocopherol + 0.4 × mg β-tocopherol + 0.1 × mg γ-tocopherol + 0.01 × δ-tocopherol + 0.3 × α-tocotrienol + 0.05 × β-tocotrienol + 0.01 × γ-tocotrienol (the formula in “VIT_E.CAL” sheet).

This makes it possible to compare the obtained data with the values from the Food Composition and Nutritional Value Tables [31] or refer to the AI in α-tocopherol equivalents. The “Total of vitamin E isoforms” row indicates the total intake of all isoforms of vitamin E.

### 2.4. Assessment of Adequecy of Vitamin E and α-Tocopherol Intake

Due to the fact that dietary reference values refer to α-tocopherol equivalents in mg/person/day or α-tocopherol in mg/person/day, the obtained results from the VitE-FFQ and the 1-day dietary record were compared for each respondent with reference values from the RDA levels (NIH) [9], and with AI values according to NIPH–NIH–NRI [13] and EFSA for the European [6] population (Table 2).

### 2.5. Statistical Analysis

Assessment of the usefulness of this tool was carried out by comparing the results of the VitE-FFQ with the results of the 1-day dietary record. Statistical analysis included the following:

- Calculation of differences between the intake levels of tocopherols and tocotrienols obtained from the two methods: the normality of the distribution of results was analyzed using the Shapiro–Wilk test, and then the Mann–Whitney U test was applied.
- Analysis of the correlations between results: the normality of distribution of the results was analyzed using the Shapiro–Wilk test, and, afterwards, Spearman’s rank correlation was applied for nonparametric distribution.
- Analysis of the Bland–Altman plots in the assessment of agreement (VitE-FFQ vs. 1-day dietary record); the results were interpreted using the Bland–Altman index, whereas the limits of agreement values (LOA) were calculated as the sum of the mean absolute difference in the consumption of individual forms of tocopherols and tocotrienols, measured by the two methods, and the ± standard deviation of the absolute difference of the assessment compounds intake recorded by the two methods and magnified by 1.96; the Bland–Altman index (%) was calculated as a percentage of persons beyond the limits of agreement (LOA). Good reproducibility of the measurement was proved by a minimum of 95% difference within the ±2 SD limits, which corresponds to the Bland–Altman index amounting to no more than 5%.

A level of significance at *p* ≤ 0.05 was accepted. Statistical analysis was carried out using Statistica software version 13.0.0. (StatSoft, Tulsa, OK, USA) and the Bland–Altman Statistica software macro by Matt Coates version 2009 (StatSoft, Inc., Tulsa, OK, USA).

## 3. Results

### 3.1. Characteristics of the Participants

A total of 447 adults were included in this study; 73% were women and 76% were people with secondary and university education. More than half of the study group (58%) lived in a city of >100,000 inhabitants. The largest share of the study sample (60%) comprised individuals aged 18–25, 62% of which were women and 52% of which were men, respectively. The smallest were the age groups of 41–60 years (10% of the total) and >60 years (1% of the total) (Table 3).

### 3.2. Intake of Tocopherols, Tocotrienols, and α-tocopherol Equivalents

The average intake of α-tocopherol equivalents in the study group was 11.3 mg based on the data computed using the FFQ method and 12.8 mg based on the 1-day dietary record (Table 4). The α- and γ-tocopherol forms had the greatest share in the total content of vitamin E (55% and 38% of the total sum of tocopherols, respectively).

The mean intake values of tocopherols, tocotrienols, their sums, and α-tocopherol equivalents calculated using these two methods did not differ significantly (*p* > 0.05) (Table 4). The dominant forms of tocopherols were α- and γ-, regardless of the data collection method. The major sources of α-tocopherol in the diet were almonds and sunflower seeds, while chips, crackers, nachos, and rapeseed oil were the major sources of γ-tocopherol. Among the tocotrienols, the β- form (49% of the total sum of tocotrienols) and α- and γ- forms (24% of the total sum of tocotrienols) dominated. Their main sources in the diet were wholemeal bread, wholegrain pasta, brown rice, and cornflakes. Tocopherols accounted for 94.3% of the total pool of consumed vitamin E, whereas tocotrienols accounted for only 5.7%.

Only 40–57% of respondents had an adequate vitamin E intake (Table 5). In more than half (55–57%), the requirement for vitamin E according to DRIs in Poland was met [13], regardless of the estimation method. The intake of α-tocopherol did not exceed the UL value for any of the respondents.

### 3.3. Analyzing the Agreement between Dietary Data Obtained by FFQ and Dietary Record Methods

**Figure 2:**
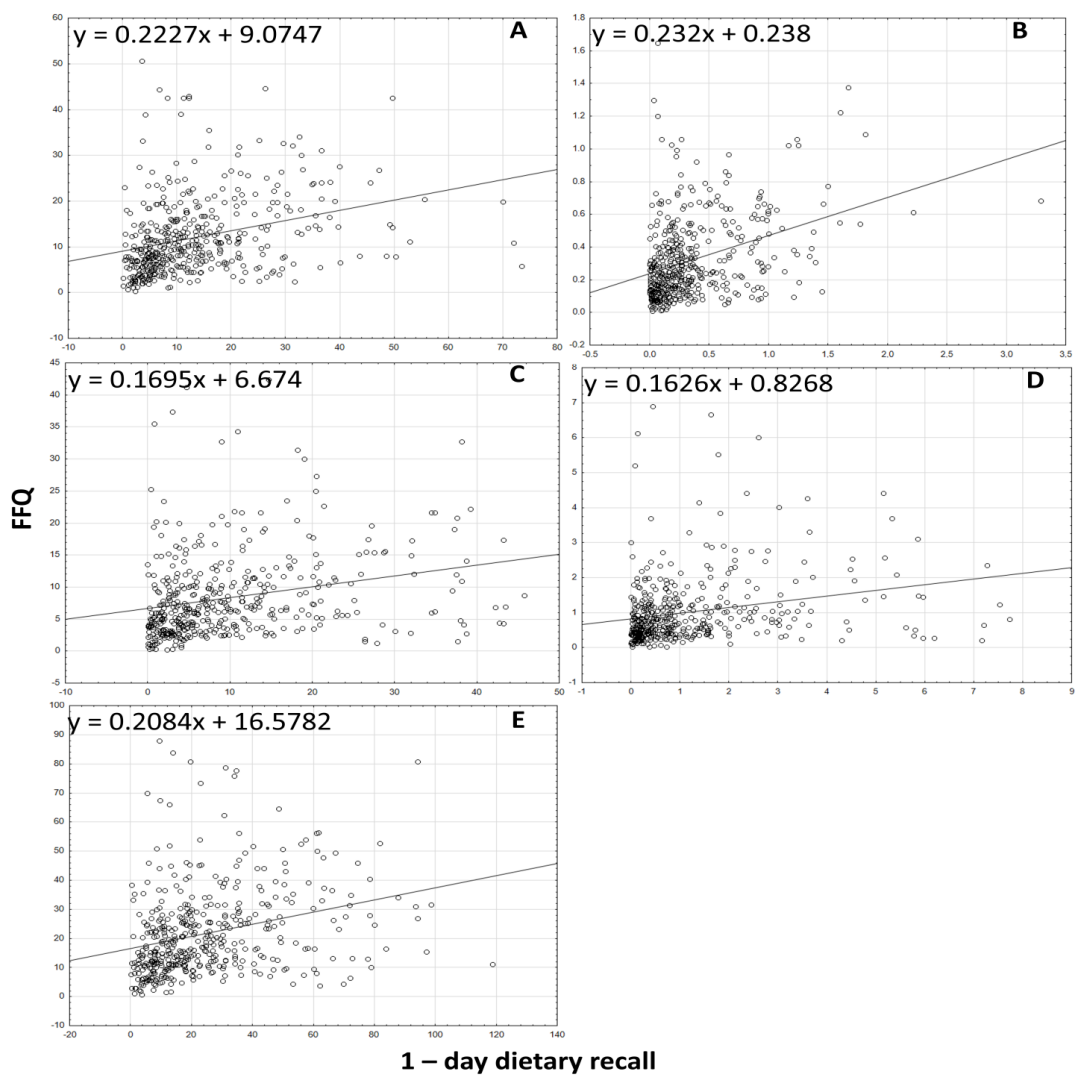
Correlation analysis between VitE-FFQ results and 1-day dietary record for daily intake of α-tocopherol (**A**), β-tocopherol (**B**), γ-tocopherol (**C**), δ-tocopherol (**D**), and sum of tocopherols (**E**).

**Figure 3:**
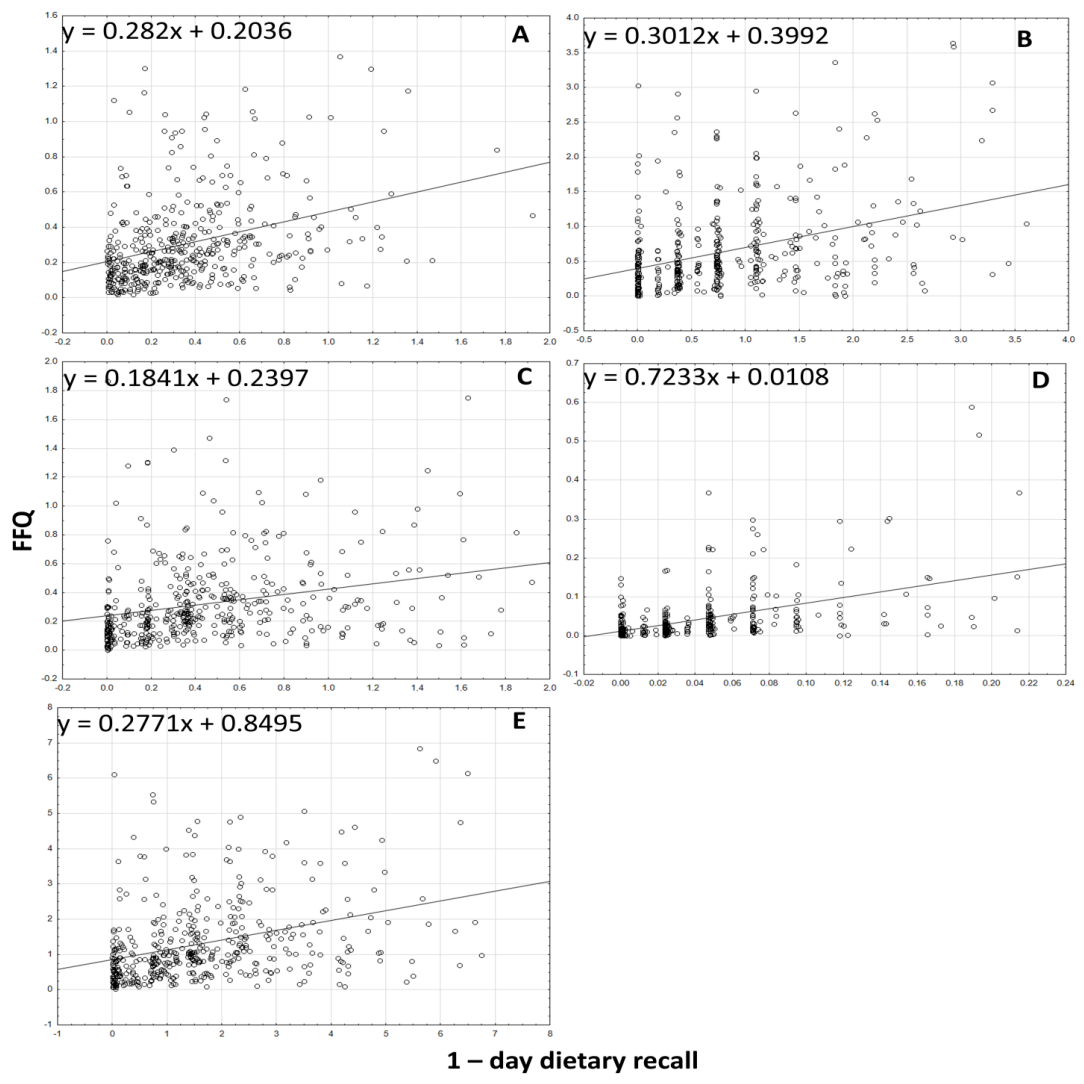
Correlation analysis between VitE-FFQ results and 1-day dietary record for daily intake of α-tocotrienol (**A**), β-tocotrienol (**B**), γ-tocotrienol (**C**), δ-tocotrienol (**D**), and sum of tocotrienols (**E**).

**Figure 4:**
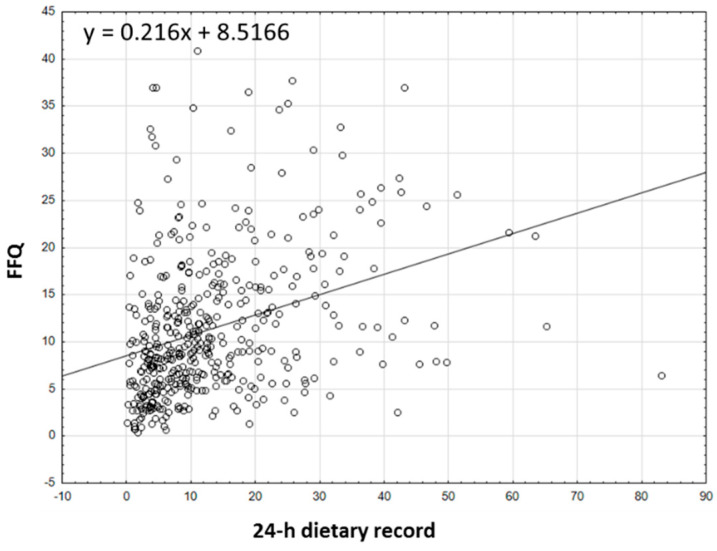
Correlation analysis between VitE-FFQ results and 1-day dietary record for daily intake of α-tocopherol equivalents.

The analysis of the correlation between the results for the intake of all forms of tocopherols and tocotrienols, the sum of tocopherols and tocotrienols, and the α-tocopherol equivalents obtained using the VitE-FFQ and the 1-day dietary record is presented in Figure 2, Figure 3 and Figure 4. Spearman’s rank correlation coefficient revealed a statistically significant association for data obtained using the VitE-FFQ and the 1-day dietary record for δ-tocotrienol (*p* < 0.01, R = 0.545), α-tocopherol (*p* < 0.01, R = 0.409), α-tocotrienol (*p* < 0.01, R = 0.405), sum of tocotrienols (*p* < 0.01, R = 0.393), α-tocopherol equivalents (*p* < 0.01, R = 0.382), β-tocotrienol (*p* < 0.01, R = 0.375), β-tocopherol (*p* < 0.01, R = 0.374), sum of tocopherols (*p* < 0.01, R = 0.367), γ-tocotrienols (*p* < 0.01, R = 0.358), γ-tocopherols (*p* < 0.01, R = 0.340), and δ-tocopherols (*p* < 0.01, R = 0.320).

**Figure 5:**
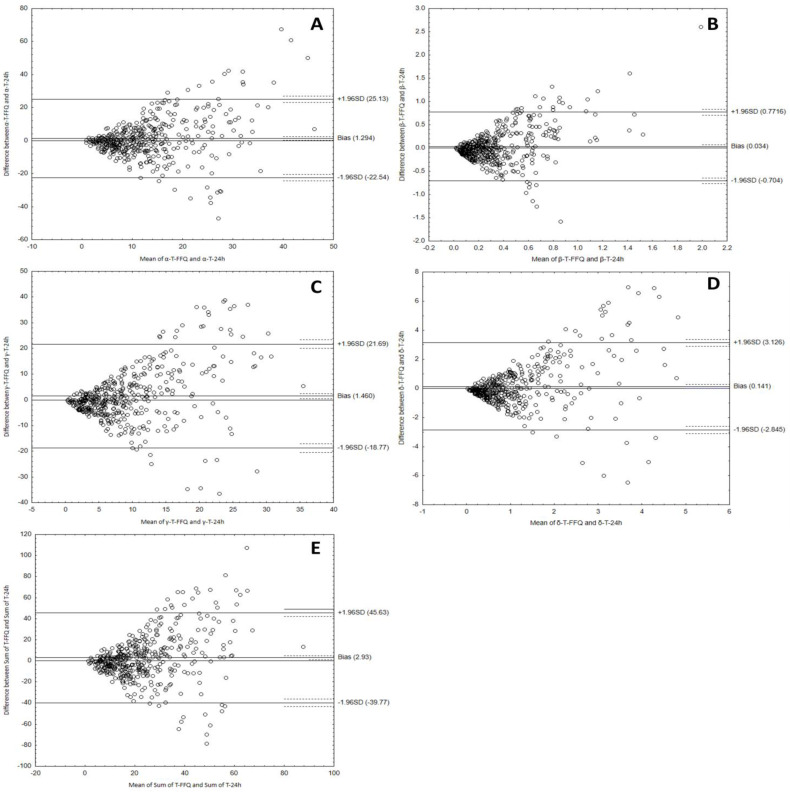
Bland–Altman plots comparing results of α-tocopherol (**A**), β-tocopherol (**B**), γ-tocopherol (**C**), δ-tocopherol (**D**), and sum of tocopherols (**E**) intake using the VitE-FFQ and 1-day dietary record.

Bland–Altman plots comparing VitE-FFQ results with a 1-day dietary record of daily tocopherol intake with diet are shown in Figure 5.

The results for the comparison of the two methods for assessing the intake of tocopherols from the Bland–Altman analysis are presented in Table 6.

**Figure 6:**
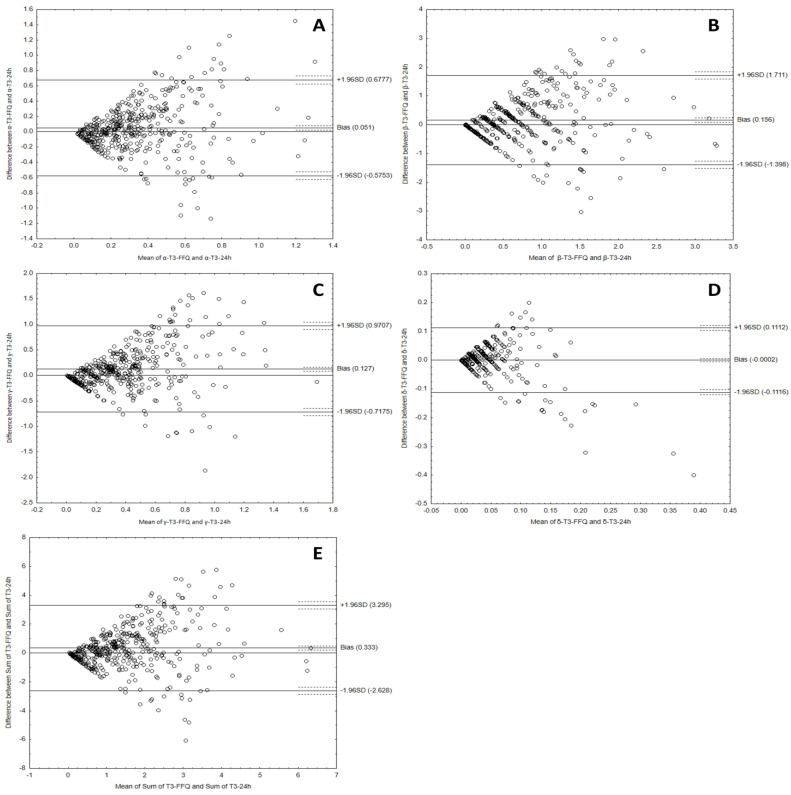
Bland–Altman plots comparing consumption data for α-tocotrienol (**A**), β-tocotrienol (**B**), γ-tocotrienol (**C**), δ-tocotrienol (**D**), and sum of tocotrienols (**E**) using the VitE-FFQ and 1-day dietary record.

The Bland–Altman plots comparing data collected with VitE-FFQ and a 1-day dietary record of daily tocotrienols intake are shown in Figure 6.

The Bland–Altman analysis results for the comparison of both methods for determining tocotrienol consumption are shown in Table 7.

**Figure 7:**
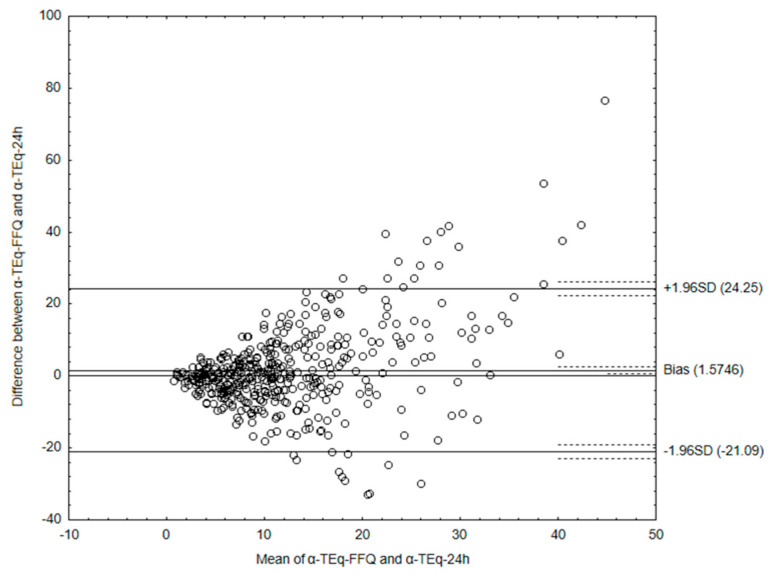
Bland–Altman plot comparing α-tocopherol equivalent intake data obtained from the VitE-FFQ and 1-day dietary record.

The Bland–Altman plot comparing data obtained from the VitE-FFQ and a 1-day dietary record of α-tocopherol equivalent intake is shown in Figure 7.

The mean absolute difference in α-tocopherol equivalent (Figure 7) intake was observed to amount to 1.57 mg. After adding ± 1.96 standard deviation for the LOA, an interval from −21.1 (lower agreement limit) to 24.25 (upper agreement limit) was obtained. The number of individuals observed to be within the LOA value was 420 out of 447, which confirmed the Bland–Altman index of 6.04%.

To sum up, the Bland–Altman indexes of less than 10% indicate a high agreement of the results regarding the consumption of tocopherols and tocotrienols, obtained using both methods and using the developed calculator, which confirms its usefulness.

## 4. Discussion

Tocopherols and tocotrienols are important biological active compounds. For this reason, the developed VIT_E.CAL may prove to be helpful in adequately assessing vitamin E and also α-tocopherol intake, in arranging a balanced diet and dietotherapy, and in the prevention of diseases. In our study group, the mean tocopherol intake was about 22–25 mg/d and the mean tocotrienol intake was about 1.3–1.6 mg/d. The dominant forms of vitamin E in the diet were α- and γ- forms (55% and 38% of the total sum) among tocopherols and β- and γ- forms (49% and 24% of the total sum) among tocotrienols. Although the mean intake of vitamin E in α-tocopherol equivalents was about 12 mg per day, which is close to the DRI, only 55% of the study group had adequate intake. Considering α-tocopherol alone, only diets in 42–45% of the group met the recommendations.

Vitamin E is a nutrient derived from a limited number of products in the Polish diet, and the richest sources of vitamin E are vegetable oils (rapeseed, sunflower, and wheat germ). In addition, it is found in some cereal products, nuts, and vegetables, mainly dark-green ones [13]. In this study, the most abundant form of vitamin E in the diet of the studied group was α-tocopherol, and its primary sources were almonds and sunflower seeds. These data are supported by results from a multicenter, randomized, double-blind, prospective intervention study that assessed, among other things, the intake of vitamin E in the diet of older Europeans [33]. The second predominant form of vitamin E in the assessed diet in this study was γ-tocopherol, and its main sources were potato chips, crackers, nachos, and rapeseed oil. It seems that this is due to the fact that these products are highly processed foods, and during their processing, for example, frying, vegetable oils are used. A rich dietary source of γ-tocopherol are plant seeds and derived products such as vegetable oils, e.g., from corn, soybean, sesame, walnuts, pecans, and peanuts [26,34]. According to Dietrich et al. [35], γ-tocopherol is the most prevalent form of vitamin E in the American diet.

The main part of tocotrienol intake comprised β- and next α- and γ- forms, but these were small amounts compared to the sum of tocopherols and tocotrienols (21.7 mg vs. 1.3 mg). Their main sources in the diet were wholemeal bread, wholegrain pasta, brown rice, and cornflakes. Research indicates that the sources of tocotrienols could also be wheat germ, barley, oats, rye, corn, and others [27]. Tocotrienols are found in significant amounts in palm oil and rice bran oil, but they are less common than tocopherols in other vegetable oils popular in the Polish local diet [19]. Comparing the obtained values with the results of other authors allows us to assess the developed tool as correct. Sookwong et al. [36,37] estimated the daily intake of tocotrienols in the Japanese population at 1.9–2.1 mg T3/day/person. Data from the Korea National Health and Nutrition Examination Survey (KNHANES) 2016–2019 showed an average daily intake of tocotrienols of 1.61 mg/person. The main known sources of tocotrienols (annatto, palm oil, and rice bran oil), widely used in Asian countries also as a health supplement, are not included in this calculator as they are not commonly found in the local diet. It seems that their consumption level in the present study is related to the reported consumption of processed products (chips, crackers, and nachos), for which palm oil is used, for example.

The results of this study showed that only 42% of the subjects consumed α-tocopherol in the amount of at least 15 mg/day and 57% in the amount of at least 8 mg vitamin E/day for women and 10 mg for men, respectively. This is in line with the results published in the Global Systematic Review of Vitamin E Intake, which indicated that in most of the 176 studies included from 46 countries, the average intake levels of α-tocopherol and the average intake of all eight vitamin E isomers were below recommended intakes in all reported countries and regions. The lowest consumption of α-tocopherol in the world was reported in the USA, Spain, Brazil, and Poland and averaged below or close to 5 mg per day [7]. According to EFSA data [6], the average consumption of α-tocopherol in European adults ranged from 7.8 to 12.5 mg/day in women and from 8.2 to 16.0 mg/day in men. In adults, the average α-tocopherol equivalent intakes ranged between 8.9 and 13.5 mg/day in females and between 10.1 and 16.0 mg/day in males. In previous studies [38,39,40,41], the intake of vitamin E by Poles was assessed as sufficient and varied from 44% to even 199% of the DRI. Notably, food consumption in these studies was reported using various types of food records. These methods are often described as burdensome for respondents and interviewers, time-consuming, and difficult [42]. Moreover, misreporting in a food record is often observed as a result of a change in eating behavior during the reporting period. Consequently, this leads to underestimation or overestimation of energy in the diet [43,44] and fat content, which is the primary source of vitamin E. Studies comparing food-recording methods noticed a problem with the underestimation of fat intake by the respondents. The OPEN study noted a trend toward lower fat intake for both men and women [45]. In the study by Goris et al. [46], obese men underestimated their fat intake. It seems that the semiquantitative FFQ method for a short period, relating to the consumption of a main source of micronutrients, might be more accurate. The FFQ is the method most commonly used to record food consumption in large-scale studies [47]. It can be used to assess the consumption of individual products, product groups, and the amount of nutrients present in food as well as to determine dietary patterns [48]. Questionnaires are defined as quantitative when they take into account the portion size of consumed products or their groups [49]. FFQs are often used in diet assessment because they are practical, easy to use, accessible, inexpensive to use, and engage the respondent, better describing the usual diet than other methods [50]. In the FFQ reproducibility study, cross-match was checked (test–retest), and the lowest cross-classification ability for fat-source products (vegetable oil, margarine, and butter blend) was noted at 63.8% [48]. These products are a source of vitamin E, which implies the need to refine the tool that will take into account such food products.

There are attempts to create and validate food frequency questionnaires taking into account tocopherols and tocotrienols or α-tocopherol equivalent intake [48,51,52,53,54,55,56].

In this study, the values of the obtained Bland–Altman indexes for all forms of vitamin E, the sum of tocopherols and tocotrienols, and the α-tocopherol equivalents were below 10%, which proves a good result of the questionnaire agreement with the 1-day dietary record. The VitE-FFQ includes the usual food products grouped to minimize the risk of respondents underestimating their consumption of fat, which is the primary source of various forms of vitamin E in the diet. Moreover, since the questionnaire was designed with typical European food products in mind, it would be relatively easy to modify. The VIT_E.CAL tool can be used as a practical, quick tool to assess the consumption of individual forms of tocopherols, tocotrienols, vitamin E, and α-tocopherol equivalents in the adult Polish population. The tool can be applied to assess vitamin E consumption among Europeans after introducing appropriate modifications. At a later stage, it is necessary to validate the developed questionnaire, preferably using the biochemical concentration of the consumed forms of vitamin E in the blood or metabolites of various forms of vitamin E, which will be the most reliable. A validated questionnaire combined with the calculator has the potential to provide a reliable intake assessment.

The results of this study should not be extrapolated to the Polish population in general because random sampling of the study group was used. The limitation of this study is the lack of assessment of the reproducibility of the FFQ questionnaire, which limits the possibilities of drawing conclusions and may increase the risk of overinterpretation of the results. A validation study is required to confirm the dependability of the obtained results. In addition, data on the content of various forms of vitamin E from the American database were used for Polish food products. The main advantage of this study is the relatively large number of respondents (n = 447), which leverages the importance and statistical power of the results. In addition, the Bland–Altman plot analysis was applied, which is considered the gold standard for validation of food intake questionnaires. However, further research on a representative Polish sample is necessary. This will allow for the extrapolation of the results on the structure of consumption of various forms of vitamin E in the country and at the same time will facilitate the assessment of the repeatability of the presented questionnaire.

## 5. Conclusions

Tocopherols and tocotrienols are important bioactive compounds and can play an important role in the prevention and treatment of many diseases, such as cardiovascular, neurodegenerative, and cancer diseases. The obtained results indicated insufficient intake of vitamin E in the study group and the need to optimize the structure of the diet in terms of the selection of sources of its individual forms. For this reason, the developed tool, VIT_E.CAL, can be used not only in arranging a balanced diet but also in diet therapy and the prevention of these diseases. Our provided database and proposed algorithm can help professionals calculate the intake of individual forms of tocopherols and tocotrienols. The use of VIT_E.CAL enables the proper assessment of vitamin E (as α-tocopherol and not α-tocopherol equivalent) in the diet of Poles, and most likely also in the European diet. The tool may also be helpful in determining the relationship between individual forms of vitamin E intake and their plasma concentrations, which in the long run may allow for the development of individualized nutritional recommendations. The obtained results indicate the need to take into account the content of individual forms of vitamin E in food/diet, which will allow for a reliable assessment of its consumption. It also seems necessary to standardize the nomenclature regarding the name of vitamin E and its use in correct nutritional assessment.

## Figures and Tables

**Table 1:** Design of the VitE-FFQ—food items, portion sizes, and household measures.

Food Product	Serving Size	Household Measures
**Vegetables**		
Broccoli/brussels sprouts/kale/asparagus/leeks	100 g	2/3 cup; 2 handfuls of leaves
Carrots/parsley root	100 g	2/3 cup
Green peas/tomatoes	100 g	2/3 cup
Beetroot/beet greens/pumpkin/red peppers/turnip	100 g	2/3 cup; 2 handfuls of leaves
Lettuce/romaine lettuce/spinach/chicory/rucola	100 g	2/3 cup; 2 handfuls
Corn	20 g	1 tablespoon
Carrot juice/multivegetable juices	200 mL	1 cup
Other vegetables	100 g	2/3 cup; 2 handfuls of leaves
**Fruit and Fruit Products**		
Kiwifruit	100 g	2/3 cup
Raspberries/blackberries/blueberries	100 g	2/3 cup
Avocado	70 g	1/2 medium piece
Other fruit	100 g	2/3 cup
**Legumes and Legume Products**		
Peas/lentils—dry seeds	15 g	1 tablespoon
Hummus	10 g	1 teaspoon
**Nuts and Oilseeds**		
Pumpkin seeds	30 g	2 tablespoons
Linseeds	30 g	2 tablespoons
Pistachios/pecans	30 g	2 tablespoons
Almonds/sunflower seeds	30 g	2 tablespoons
Almond drink	200 mL	1 cup
Hazelnuts	30 g	2 tablespoons
Peanuts	30 g	2 tablespoons
Pine nuts	30 g	2 tablespoons
Other nuts, e.g., walnuts, cashews	30 g	2 tablespoons
**Fats**		
Canola oil	10 g	1 tablespoon
Sunflower oil	10 g	1 tablespoon
Olive oil	10 g	1 tablespoon
Grape-seed oil	10 g	1 tablespoon
Peanut oil	10 g	1 tablespoon
Wheat germ oil	10 g	1 tablespoon
Soybean oil	10 g	1 tablespoon
Coconut oil	10 g	1 tablespoon
Margarine	10 g	1 tablespoon
Mayonnaise	10 g	1 tablespoon
Pesto	10 g	1 teaspoon
Eggs	50 g	1 piece
**Cereals**		
Wholemeal bread, whole wheat pasta/brown rice/cornflakes	bread 35 g or 75 g other	1 slice or cup of dry product
**Fish and Fish Products**		
Fresh fish, e.g., salmon, trout, mackerel, herring	100 g	1 piece
Canned fish in oil, e.g., mackerel, tuna, herring, sardines	150 g	1 medium can
Other fish, e.g., canned fish in water/in tomatoes, flounder, carp, fish fingers	150 g	1 medium can
**Snacks and Others**		
Dark chocolate	5 g	1 cube
Chips/crackers/nachos	10 g	1 handful

**Table 2:** Adult dietary reference intakes (DRIs) for α-tocopherol (α-T) and α-tocopherol equivalents (α-T Eq) by different institution.

	NIPH–NIH–NRI		NIH		EFSA	
Vitamin E	α-T Eq [mg/d]		α-T [mg/d]		α-T [mg/d]	
DRIs	AI	UL	RDA	UL	AI	UL
Men	10	300	15	1000	13	300
Women	8				11	

NIPH–NIH–NRI—National Institute of Public Health–National Institute of Hygiene–National Research Institute; NIH—US National Institute of Health; EFSA—European Food Safety Authority; Eq—equivalents; AI—adequate intake; UL—upper level intake; RDA—recommended dietary allowances (based on: [6,9,13]).

**Table 3:** Characteristics of the participants.

Variables	Total *n* = 447	Women *n* = 327 (73%)	Men *n* = 120 (27%)
Age group, %			
18–25 years	60	62	52
26–40 years	29	27	32
41–60 years	10	9	15
>60 years	1	2	1
Education, %			
Primary and vocational	3	18	5
Secondary	24	7	25
University	52	53	48
While studying	21	22	22
Place of living, %			
Village	21	20	25
City < 100,000 inhab.	21	21	22
City > 100,000 inhab.	48	50	41
City > 500,000 inhab.	10	9	12
Anthropometrics (mean ± SD)			
Height, cm	171 ± 9.0	166.2 ± 6.0	181.1 ± 6.4
Body weight, kg	70.3 ± 33.2	65.1 ± 36.0	84.6 ± 16.9
BMI	24.1 ± 11.3	23.5 ± 12.8	25.8 ± 5.2

**Table 4:** Comparison of tocopherols, tocotrienols, their sums, and α-tocopherol equivalents intake (mg/day) estimated by VitE-FFQ and 1-day dietary record methods.

Vitamin E Isoforms	Methods						*p* *
	VitE-FFQ (mg/day)			1-Day Dietary Record (mg/day)			
	Mean ± SD	Median	Min.–Max.	Mean ± SD	Median	Min.–Max.	
α-T	12.0 ± 8.5	9.7	0.4–50.6	13.3 ± 11.7	10.1	0.2–73.5	NS
β-T	0.3 ± 0.2	0.2	0.1–1.6	0.3 ± 0.4	0.2	0.1–3.3	NS
γ-T	8.3 ± 6.6	6.5	0.2–42.3	9.8 ± 9.8	6.3	0.1–45.7	NS
δ-T	1.1 ± 0.9	0.7	0.1–6.9	1.1 ± 1.4	0.6	0.1–7.7	NS
Sum of Ts	21.7 ± 15.1	17.7	0.7–87.9	24.6 ± 20.5	18.3	0.2–118.7	NS
α-T3	0.3 ± 0.2	0.2	0.1–1.4	0.4 ± 0.3	0.3	0.1–1.9	NS
β-T3	0.6 ± 0.6	0.4	0.1–3.6	0.8 ± 0.7	0.7	0.0–3.6	NS
γ-T3	0.3 ± 0.3	0.2	0.1–1.9	0.5 ± 0.4	0.4	0.0–1.9	NS
δ-T3	0.1 ± 0.1	0.1	0.0–0.6	0.1 ± 0.1	0.1	0.0–0.2	NS
Sum of T3s	1.3 ± 1.2	1.0	0.1–6.8	1.6 ± 1.4	1.4	0.1–6.7	NS
α-T Eq	11.3 ± 7.6	9.2	0.4–40.9	12.8 ± 11.6	9.5	0.1–83.0	NS

* U Mann–Whitney test; FFQ—food frequency questionnaire; SD—standard deviation T/s—tocopherol/s; T3/s—tocotrienol/s; Eq—α-tocopherol equivalents; NS—not significant.

**Table 5:** Assessment of adequacy of intake of α-T and α-T equivalents.

DRIs		Percentage (%) of Individuals According to Method	
		FFQ	1-D
		*n* = 447	*n* = 447
α-T; RDA (NIH)	Adequate intake	42	45
	Inadequate intake	58	55
α-T equivalents; AI (NIPH–NIH–NRI)	Adequate intake	57	55
	Inadequate intake	43	45
α-T equivalents; AI (EFSA)	Adequate intake	40	47
	Inadequate intake	60	53
α-T equivalents; UL	Excessive intake	0	0

DRIs—daily recommended intakes; FFQ—food frequency questionnaire; 1-D—1-day dietary record.

**Table 6:** Bland–Altman results for tocopherols.

Vitamin E Isoforms	Mean Absolute Difference (mg)	Lower LOA	Upper LOA	Number of Individuals beyond the LOA	Bland–Altman Index (%)
α-tocopherol	1.29	−22.54	25.13	422 out of 447	5.6
β-tocopherol	0.034	−0.704	0.772	413 out of 447	7.6
γ-tocopherol	1.46	−18.77	21.69	416 out of 447	6.9
δ-tocopherol	0.141	−2.845	3.126	420 out of 447	6.04
Sum of tocopherols	2.93	−39.77	45.63	415 out of 447	7.2

LOA—limits of agreement.

**Table 7:** Bland–Altman results for tocotrienols.

Vitamin E Isoforms	Mean Absolute Difference (mg)	Lower LOA	Upper LOA	Number of Individuals beyond the LOA	Bland–Altman Index (%)
α-tocotrienol	0.051	−0.5753	0.6777	415 out of 447	7.2%
β-tocotrienol	0.156	−1.398	1.711	412 out of 447	7.8%
γ-tocotrienol	0.127	0.7175	0.9707	415 out of 447	7.2%
δ-tocotrienol	0.0002	−0.1116	0.1112	417 out of 447	6.7%
Sum of tocotrienols	0.333	−2.628	3.295	415 out of 447	7.2%

LOA—limits of agreement.

## Data Availability

Not applicable.

## Supplementary Materials

The following supporting information can be downloaded at: https://www.mdpi.com/article/10.3390/nu15173759/s1, VIT_E.CAL.
